# Bacterial reduction and temperature increase of titanium dental implant models treated with a 445 nm diode laser: an in vitro study

**DOI:** 10.1038/s41598-024-68780-2

**Published:** 2024-08-05

**Authors:** Markus Ahrens, Melanie Spörer, Herbert Deppe, Lucas M. Ritschl, Petra Mela

**Affiliations:** 1 Chair of Medical Materials and Implants, Department of Mechanical Engineering, TUM School of Engineering and Design, Munich Institute of Biomedical Engineering; Munich Institute of Integrated Materials, Energy and Process Engineering, Technical University of Munich, Munich, Germany; 2 Department of Oral and Maxillofacial Surgery, TUM School of Medicine, Technical University of Munich, Munich, Germany

**Keywords:** Dentistry, Dental implants, Peri-implantitis, 445 nm diode laser, Implant temperature, Decontamination, Dental diseases, Dentistry, Dental implants

## Abstract

In this in vitro study, the use of a 445 nm diode laser was investigated for the decontamination of titanium dental implants. Different irradiation protocols and the effect of repetitive laser irradiation on temperature increase and decontamination efficacy were evaluated on titanium implant models. An automated setup was developed to realize a scanning procedure for a full surface irradiation to recapitulate a clinical treatment. Three irradiation parameter sets A (continuous wave, power 0.8 W, duty cycle (DC) 100%, and 5 s), B (pulsed mode, DC 50%, power 1.0 W, and 10 s), and C (pulsed mode, DC 10%, power 3.0 W, and 20 s) were used to treat the rods for up to ten consecutive scans. The resulting temperature increase was measured by a thermal imaging camera and the decontamination efficacy of the procedures was evaluated against *Escherichia coli* and *Staphylococcus aureus,* and correlated with the applied laser fluence. An implant’s temperature increase of 10 °C was set as the limit accepted in literature to avoid thermal damage to the surrounding tissue in vivo. Repeated irradiation of the specimens resulted in a steady increase in temperature. Parameter sets A and B caused a temperature increase of 11.27 ± 0.81 °C and 9.90 ± 0.37 °C after five consecutive laser scans, respectively, while parameter set C resulted in a temperature increase of only 8.20 ± 0.53 °C after ten surface scans. The microbiological study showed that all irradiation parameter sets achieved a complete bacterial reduction (99.9999% or 6-log_10_) after ten consecutive scans, however only parameter set C did not exceed the temperature threshold. A 445 nm diode laser can be used to decontaminate dental titanium rods, and repeated laser irradiation of the contaminated areas increases the antimicrobial effect of the treatment; however, the correct choice of parameters is needed to provide adequate laser fluence while preventing an implant’s temperature increase that could cause damage to the surrounding tissue.

## Introduction

Approximately 12–18 million dental implants are inserted annually worldwide^1,2^ with a mean prevalence of peri-implantitis of 22% (95% confidence interval: 14–30%)^3^, which can be attributed to bacterial contamination^4^. Various methods, including mechanical debridement, disinfection with chemotherapeutic agents, and laser therapy are used to decontaminate dental implants^5^. Laser irradiation can have a bactericidal effect without changing the morphology of the implant surface, if used correctly^6,7^. Different laser systems are used in the clinic for periodontics and peri-implantitis therapy, including carbon dioxide (CO_2_) lasers, solid state lasers, such as neodymium-doped yttrium aluminum garnet (Nd:YAG) lasers and erbium-doped YAG (Er:YAG) lasers, and diode lasers^8,9^. The effect of diode lasers on microorganisms can be divided into photo-thermal (PT) and photo-dynamic (PD) inactivation, with PD requiring photosensitizers, like methylene blue^10^, riboflavin^11^ or hydrogen peroxide^12^, which form reactive oxygen species upon irradiation that inactivate bacteria^13^. PT therapy, on the contrary, relies solely on the damage to the bacteria caused by the laser energy^11,12^, known as phototoxicity^14–16^. Here, the associated temperature increase has to be taken into account with respect to the risk of thermal damage to the implant and the surrounding bony and soft tissue, with a 10 °C-increase being the generally accepted safety limit^17–19^. To mitigate this risk, recently, a new diode laser with a wavelength of 445 nm has been introduced and evaluated^2,18,20^. Böcher et al.^10^ showed that direct irradiation of titanium rods, contaminated with common pathogenic periodontal bacteria, resulted in a bacterial reduction of 95.03%, however no effect on the temperature increase was reported.

On the other hand, we and others^21–23^ looked at the temperature increase upon treatment of dental implants with a 445 nm diode laser and showed the importance of selecting the treatment parameters to stay within the 10 °C-limit, but did not investigate the antimicrobial efficacy of the treatment.

Lack of information on both concomitant effects may limit the correct use of the laser and eventually jeopardize its adoption in the clinical practice.

Furthermore, there is no scientific evidence, nor consensus in the field on the level of decontamination that should be reached for an effective treatment. Although bacterial reductions of 40%^24^ or 68%^25^ are commonly reported as successful, other authors indicate a 3-log (99.9%) as necessary^26–28^ and show clinical insufficiency of a 2-log (99%) reduction with a 660 nm diode laser treatment because of the high remaining bacterial count^29^. In other contexts, a minimum reduction of 3 log is often required to claim bactericidal activity^30–32^, disinfection of wounds using antiseptics and hygienic hand washing (DIN EN 13727).

Therefore, our study was designed to investigate whether a direct 445 nm laser irradiation is sufficient to remove at least 99.9% of the bacteria from a dental implant with a temperature increase below the 10 °C-threshold.

We selected irradiation parameter sets we had previously identified in a systematic study that had evaluated the temperature response curves of five different dental implant systems to a large range of 445 nm diode laser’s settings (power, exposure time, duty cycle, contact or non-contact mode). The study had showed that the laser is not inherently safe for the decontamination of ailing implants and had provided parameter sets considered suitable for the treatment because of the contained (< 10 °C) temperature change they elicited^22^. However, these had been determined with a single spot irradiation, which is not representative of a clinical procedure to treat an infected implant. Therefore, we here developed an automated scanning set-up to ensure the irradiation of the entire implant model’s surface contaminated with *Escherichia coli* (*E. coli*) or *Staphylococcus aureus* (*S. aureus*). The laser’s decontamination efficacy was evaluated as a function of the number of surface scans, and was correlated with the laser’s fluence.

## Materials and methods

### Dental laser

A SIROLaser Blue (Sirona Dental Systems GmbH, Bensheim, Germany) with a wavelength of 445 ± 5 nm was used for the irradiation experiments. The system can be operated in either a continuous (cw) or pulsed (p) mode (chopped mode) with a power range from 0.2 to 3.0 W. The laser light was transmitted by a flexible quartz glass fiber with a core diameter of 320 µm.

### Samples

Cylindrical titanium specimens (Dentsply Sirona Deutschland GmbH, Bensheim, Germany) with a diameter (D) of 5.5 mm and a height of 17.0 mm were used as implant models, resembling dental implants in terms of material and surface finish. The samples were sterilized by autoclaving at 121 °C for 20 min (Laboklav 25 V, SHP Steriltechnik AG, Detzel Schloss, Germany).

### Experimental setup

**Figure 1:**
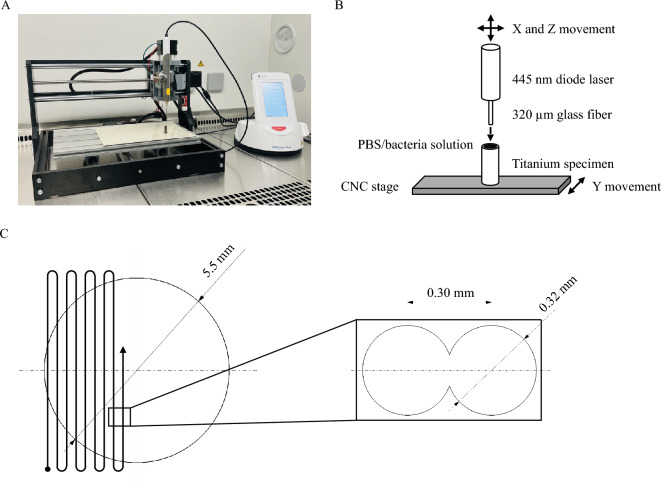
Experimental setup. CNC 3018 Pro system with the clamped handle of the SIROLaser Blue (**A**); schematic representation of the experimental setup (**B**); meandering scanning path of the laser and overlap of the irradiated circular areas (**C**).

An automated laser scanning setup was built, based on a computerized numerical control (CNC) system (CNC 3018 Pro Engraver, Guangzhou Lingyue Electronic Technology Co., Ltd., Guangzhou, China) and endstops (Muzoct M-MK-144, Shenzhen MengFeiHang E-Commerce Co., Ltd., Shenzhen, China) (Fig. 1A and B). The CNC system had an accuracy of 0.08–0.10 mm and was controlled via the software Candle (Grblcontrol Candle 1.1.7, open-source software), which implements G-code. The code ensured that the laser scanned the implant’s surface in a meandering pattern (Fig. 1C), resulting in irradiation of the complete surface. The traverse path was set such that the irradiated circular areas of the laser overlapped by 0.02 mm (Fig. 1C).

### Laser and scanning parameters

Three different irradiation parameter sets were used for the laser treatment of the titanium rods according to Deppe et al.^22^: A (cw mode, 0.8 W, and 5 s), B (p mode, DC 50%, 1 W, and 10 s), and C (p mode, DC 10%, 3 W, and 20 s). The surfaces of the titanium rods were scanned 1, 2, 3, 4, 5, and 10 times with each set of laser parameters. For multiple scans, the laser immediately returned to the starting position without irradiating the rod and started irradiation again from this position. The mode of the laser (cw or p), the duty cycle (DC), the power (P), the irradiation time (t), the distance between the specimen surface and the glass fiber (d), the scanning speed (v) and the fluence (H) are specified for each parameter set in Table 1. The average laser fluence (H) was calculated over the whole surface using Eq. (1):

1 $$H(n)=\frac{DC*P*n*t}{{\left(\frac{D}{2}\right)}^{2}*\pi } \left[\frac{J}{{cm}^{2}}\right]$$

where D is the sample’s diameter and n the number of scans.

**Table 1:** Irradiation parameter sets A, B, and C: mode of the laser, duty cycle (DC), power (P), irradiation time (t), distance between the specimen surface and the glass fiber (d), scanning speed (v) and fluence (H) for a single scan.

Parameter set	Mode	DC [%]	P [W]	t [s]	d [mm]	v [mm/min]	H [J/cm^2^]
A	cw	100	0.8	5	2	948	16.8
B	p	50	1	10		474	21.0
C	p	10	3	20		237	25.3

### Temperature measurements

A thermal imaging camera (FLIR E60, FLIR® Systems, Inc., Wilsonville, Oregon, USA) was used to measure the temperature of the titanium rods immediately before and at the end of the laser irradiation. The emission coefficient ε for the titanium specimens was determined using comparative measurements at room temperature and 37 °C as recommended by the company and was determined as 0.99 ± 0.01 (n = 3). The temperature was measured in correspondence of a point in the middle of the irradiated surface using the software FLIR tools (FLIR® Systems, Inc., Wilsonville, Oregon, USA).

### Microbiological investigations

The effect of the 445 nm diode laser was tested on implant models contaminated with Gram-negative *Escherichia coli* (*E. coli*, DSM 498) and Gram-positive *Staphylococcus aureus* (*S. aureus*, NCTC 8325). 100 µl of bacterial suspension stored at -86 °C was thawed in 900 µl of phosphate-buffered saline (PBS) and adjusted to an optical density (OD) of 0.15–0.20 using PBS (OD_620_, Multiskan FC, Thermo Fisher Scientific Inc., Waltham, Massachusetts, USA). A volume of 5 µl of the bacterial suspension was applied to the surface of the specimen. Irradiation occurred within 1 min after contamination with the parameters referred to in Table 1 for both bacteria types. After irradiation, the specimens were transferred to 4.5 ml of PBS-Tween™ 80 solution (0.1%vol) and vortexed for 30 s. Dilution steps (10^0^–10^−3^) of the bacterial suspension were prepared by automatic plating equipment (easySpiral Dilute®, Interscience, Saint Nom la Brétèche, France), and 50 µl of each dilution step was plated onto 90 mm agar plates in exponential mode. Incubation was performed at 37 °C for 24 h for *E. coli* and 48 h for *S. aureus,* and the colony forming units (CFU) were counted by using a colony counter (Scan 500, Interscience, Saint Nom la Brétèche, France). The decontamination efficacy of the laser was determined by the logarithmic (log) reduction *R*_*X*_ of the colony-forming units, which was calculated using Eq. (2):

2 $${R}_{X}={\text{log}}_{10}\left(\frac{{CFU}_{Y}}{{CFU}_{X}}\right)$$

where ${CFU}_{X}$ is the number of CFU of the sample and ${CFU}_{Y}$ is the number of CFU of the reference. A log reduction of at least 3, which corresponds to a reduction of 99.9%, was defined as the minimum value for successful decontamination. Not laser irradiated and contaminated specimens served as controls and were transferred directly into the PBS-Tween™ 80 solution.

### Statistical analysis

All experiments were performed in triplets, and data is presented as mean ± standard deviations. The temperature increase as a function of the number of scans, as well as the logarithmic reduction of bacteria as a function of the number of scans, were statistically analyzed. Normal distribution of the data and the homogeneity of variance were first checked by Kolmogorov–Smirnov (α = 0.05) and F-test (α = 0.05). A two-way ANOVA was performed, and a *p*-value < 0.05 was considered statistically significant. Statistical analyses were performed using GraphPad Prism 10 (version 10.0.3 (275), GraphPad Software, San Diego, CA, USA) and MS Excel 2019 (version 1808, Microsoft Corporation, Redmond, WA, USA).

## Results

### Temperature measurements

**Figure 2:**
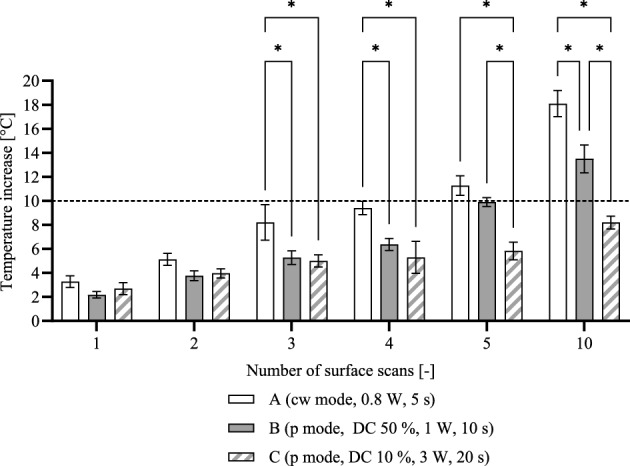
Temperature increase on the surface of the titanium sample by the laser treatment as a function of the number of surface scans. Data given as mean ± standard deviation (n = 3). The dashed line represents a critical temperature increase of 10 °C. Asterisks mark statistically significant differences based on a *p*-value of 0.05.

The irradiation of the titanium specimens led to a homogeneous temperature distribution on the surface, and the temperature increase due to laser irradiation is shown in Fig. 2. For all irradiation parameter sets A, B, and C, a temperature rise of less than 10 °C was observed for a single irradiation. Repeated irradiation of the specimens resulted in a steady increase in temperature, with protocol C showing the smallest temperature increase and remaining below the critical threshold of 10 °C even after ten scans of the surface. For parameter sets A and B, a temperature increase of around 10 °C or higher occurred after five surface scans.

### Microbiological investigations

**Figure 3:**
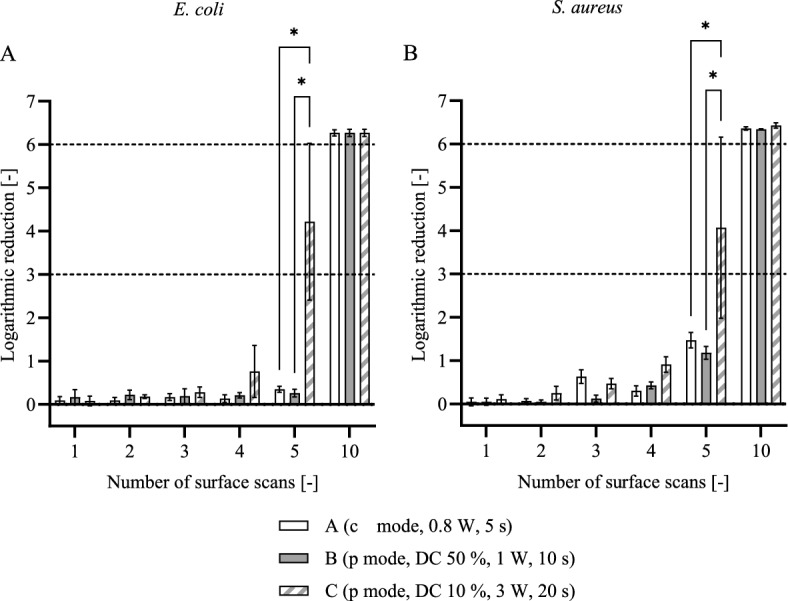
Laser treatment of contaminated titanium implant models. Log reduction of *E. coli* (**A**) and *S. aureus* (**B**) as a function of the number of surface scans. Data given as mean ± standard deviation (n = 3). The dashed lines represent a 3-log (99.9%) and a 6-log (99.9999%) reduction in bacteria. Asterisks mark statistically significant differences based on a *p*-value of 0.05.

Figure 3 shows the log reduction of *E. coli* and *S. aureus* as a function of the number of surface scans. For the irradiation parameter sets A and B, no log reductions higher than 0.26 ± 0.09 for *E. coli* and 1.18 ± 0.15 for *S. aureus* could be obtained with a temperature increase lower than 10 °C. Parameter set C resulted in a reduction higher than 3 log (i.e. 99.9%) for both bacterial strains after five scans, although with a high variation, while a complete bacterial reduction (6-log or 99.9999%) was achieved after ten surface scans for all protocols.

## Discussion

Laser treatment of an implant is associated with an increase in temperature that may be harmful to the surrounding tissue, with a change of 10 °C above body temperature, for a maximum of 1 min, being generally considered a safety threshold^17–19^.

**Figure 4:**
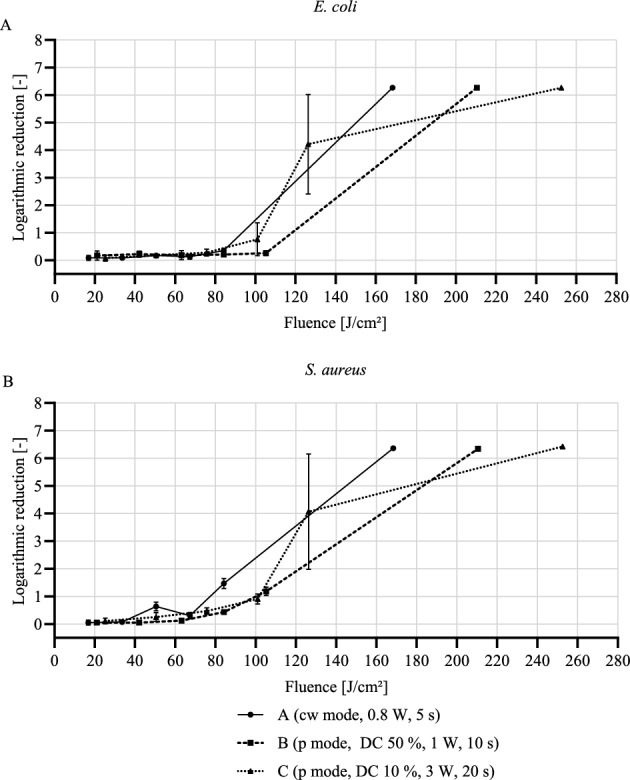
Bactericidal effect of a 445 nm laser. Log reduction of *E. coli* (**A**) and *S. aureus* (**B**) as a function of the applied average laser fluence. Data given as mean ± standard deviation (n = 3).

Irradiation with the parameter sets A, B, and C were well within this limit for a single scan of the titanium specimens in agreement with Deppe et al.^22^, who used exactly the same sets, however, differently from this study, had a fixed spot irradiation. The distribution of the laser energy while scanning over the whole surface and the resulting faster heat transfer to the environment can explain the smaller increases in the temperature we obtained. With repeated scans there was a further rise in temperature with differences for the used parameter sets becoming more evident at high number of scans, specifically set A resulted in temperature increases significantly higher than those associated to sets B and C. This could be explained by the respective laser irradiation duration, with longer times facilitating heat transfer to the surroundings. Consequently, even though set C had the highest fluence, it caused the smallest temperature increases, staying well below the 10 °C-limit even for 10 scans. For parameter set A, only a maximum of four and for parameter set B, only a maximum of five scans of the surface could be performed below this limit. Under these conditions, only a weak decontaminating effect was observed for both *E. coli* and *S. aureus* with bacterial reductions in average lower than 50% and with a maximum of 93% (1.18 log) only for *S. aureus* after 5 scans with parameter set B. Bombeccari et al. showed that a 93.6% reduction due to a PD therapy with a diode laser resulted in a recolonization of some bacteria species after 6 months^33^. There is no generally accepted minimum requirement for the percentage reduction of a treatment. In literature, the effectiveness of laser treatment for contaminated implants is often assessed based on criteria such as plaque index, gingival recession, probing pocket depth, clinical attachment level, bone loss, and bleeding on probing^34,35^, and it is not always reported whether a sufficient reduction in bacteria was achieved, with results ranging from 30^36^ to 100%^37^. Therefore, in the present study the effect of laser treatment was considered antimicrobial if there was a bacterial reduction of at least 3 log steps (99.9% reduction of bacteria), which is the minimum requirement for disinfection^30,38^, while from a clinical point of view a 6-log reduction (99.9999%) has a higher relevance^13,29^. Only irradiation with parameter set C reached both requirements after 5 and 10 consecutive scans, respectively, while inducing a temperature increase lower than 10 °C. This is likely due to the high peak power of the laser (3 W), combined with the low duty cycle (10%) and the long treatment time (20 s), preventing excessive heating of the sample. This confirms that the bacterial reduction was not due to the increase in the temperature but to the phototoxicity of the laser irradiation^14–16^, in agreement with Schoop et al.^39^, who showed that there was no significant relation between the temperature increase and the decontamination effect on slices of root dentin treated with an Er:YAG laser. Indeed, in our study, similar temperature increases, obtained with different parameter sets, corresponded to very different log reductions. Various studies showed that bacteria, including *E. coli* and *S. aureus*, can be inactivated by irradiation with blue light (400–470 nm) emitted by light-emitting diodes^15,40^, super luminous diodes^41,42^, diode laser (450 nm)^43^, or xenon light source^44^, with the highest decontamination effect obtained at shorter wavelengths^41,44^ and in a dose dependent manner^41,42^. Still, some of these studies did not reach a 3-log reduction^41–43^. Our results show that a certain fluence was required to have an antimicrobial effect and that, therefore, higher scan numbers were needed. Figure 4 shows the log reduction for *E. coli* and *S. aureus* as a function of the fluence of the 445 nm diode laser. Parameter set C yielded an average 4.07 ± 1.71 log-reduction after five scans and a fluence of 126 J/cm^2^, although with a high variability. This fluence value is much higher than those used in other studies (e.g., max 15 J/cm^2^ in Guffey et al.^41^, max 24 J/cm^2^ in de Sousa et al.^43^, and max 60 J/cm^2^ in Enwemeka et al.^42^), where, however, only weak bactericidal effects were shown, with reductions lower than 2 log steps. Repeated scans have the direct effect of increasing the fluence but also of improving the energy distribution on the surface because of the small machine-related offset of the laser starting position with each scan. Considering the incomplete surface irradiation within a single scan for the pulsed mode with a duty cycle of 10%, the offset results in an increased area coverage. After 10 scans a 6-log reduction was achieved. This was the case also for sets A and B, even with lower fluences, however with temperatures largely above the safety threshold. An incomplete surface irradiation seems to have been critical also in the study by Huang et al. in which contaminated titanium disks were irradiated with an Er:YAG laser with a wavelength of 2.94 µm following a meandering pattern^45^. By having a laser spot size of 0.6 mm, a 1 mm line interdistance and a single pass, the authors did not create an overlap between the irradiated lines. This can be appreciated from the fluorescence staining images in which no continuous, but rather spot-like removal of the bacteria was shown, with an overall reduction of only 84.1% (< log 1).

Overall, the biologicasl experiments showed that *S. aureus* was more sensitive to laser treatment than *E. coli*, which could be due to the difference in cell wall structure between Gram-negative and Gram-positive bacteria^13,39^ and is in agreement with other studies showing bactericidal effect dependent on the nature of the treated bacteria^40,43,44^.

Although we implemented a scanning procedure to create an experimental condition similar to a clinical one, our in vitro set-up does not account for the difference in heat transfer between implant and air or surrounding tissue. Although it can be argued that an implant cools faster after laser treatment when it is surrounded by tissue, we deemed it important to stay within the 10 °C safety limit. The temperature obtained with the parameter set C when yielding a 6-log reduction was well below the threshold, in line with Gutknecht et al. that suggested that a temperature rise even lower than 10 °C could be considered as limit in in vitro experiments^46^. Furthermore, the presence of different fluids such as blood and saliva in vivo might influence the efficiency of the irradiation, although it could be argued that the liquid component of the bacterial suspension only minimally attenuated the laser^43^.

For the clinical suitability of a 445 nm laser for implant decontamination, the effect of the irradiation on the surrounding tissue should be considered. Here in vitro studies showed contradictory results^47^ such as opposite effects of irradiation on the viability of the same type of cells and inconsistent effects of equivalent laser fluences on cell viability. Szymański et al.^48^ reported a dose-dependent statistically significant reduction in fibroblasts (BJ-5ta) following 445 nm-irradiation with fluences higher than 50.5 J/cm^2^, whereas no decrease in viability of keratinocytes (hTERT-immortalized) was observed even at fluences as high as 204.1 J/cm^2^. However, Rupel et al.^49^ showed that direct 445 nm irradiation with high fluences of 120 J/cm^2^ killed immortalized human skin keratinocyte cells (HaCaT), while no effect on the viability of human oral mucosa epithelial cells (TR146) was detected. The authors suggested the different origin of the cells (skin vs oral mucosa) or their transformation grades (immortalized vs. neoplastic) as possible reasons. In another study, Motlagh et al.^50^ demonstrated that 445 nm irradiation of gingival fibroblasts with only 4 J/cm^2^ reduced the cell proliferation and caused cell necrosis. The conflicting data regarding the impact of a 445 nm laser on cells in in vitro studies and the fact that these studies do not recapitulate the regenerative potential of native tissues, underscore the need for an in vivo evaluation of the clinical applicability of the 445 nm laser for decontaminating dental implants.

## Conclusion

In this study, the bactericidal effect of a 445 nm diode laser was demonstrated against *S. aureus* and *E. coli*. Parameter set C (p mode, DC 10%, 3 W, and 20 s) yielded a bacterial reduction of 6 log (99.9999%) after 10 surface scans, while remaining within the 10 °C temperature limit. Therefore, irradiation with a 445 nm diode laser can be considered as an alternative treatment option for peri-implantitis, provided that appropriate laser parameters are selected to obtain an adequate fluence.

## Data Availability

The datasets of the study are available from the corresponding author upon reasonable request.
